# Successful nasal intubation with a laryngeal nerve monitoring tube using bronchoscopy in a patient with plunging goiter: a case report

**DOI:** 10.1186/1756-0500-6-467

**Published:** 2013-11-15

**Authors:** Savino Spadaro, Manuela D’Agata, Marco Verri, Riccardo Ragazzi, Carlo A Volta

**Affiliations:** 1 Department of Morphology, Surgery and Experimental Medicine, Section of Anaesthesia and Intensive Care, S. Anna Hospital, University of Ferrara, via Aldo Moro 8 Cona, Ferrara, FE 44124, Italy

**Keywords:** Difficult airway, Recurrent laryngeal nerve monitoring, Fiberoptic bronchoscopy, Thyroid surgery

## Abstract

**Background:**

The appropriate positioning of nerve integrity monitoring during thyroid surgery is of relevance. In this case report we describe our experience with accurate placement of a nerve integrity monitoring endotracheal tube, obtained by fiberoptic control, in a patient with expected difficult airway management.

**Case presentation:**

We report the case of a 70-year-old obese woman scheduled for elective total thyroidectomy due to plunging intrathoracic goiter. The preoperative indirect laryngoscopy pointed out a massive bombè of the hypopharyngeal wall to the right and right vocal cord paralysis. The epiglottis was oedematous and the glottis could not be identified. On physical examination, the tongue was large and a Mallampati’s score of 3 was determined. Hence, due to an expected difficult airway management, a nasal intubation with an electromyographic nerve integrity monitoring endotracheal tube trough fiberoptic bronchoscopy was successfully performed.

**Conclusion:**

Our experience suggests that nasal intubation can be safely performed by using a nerve integrity monitoring tube with the help of fiberoptic bronchoscopy.

## Background

Nasal fiberoptic intubation is indicated for patients in whom oral intubation is known to be or is expected to be difficult [1]. Whenever possible, an 7–7.5 mm endotracheal tube should be used for nasal intubation because it has an acceptable flow resistance and it is large enough to allow bronchoscopy [2]. The use of a nerve integrity monitoring electromyographic endotracheal tube (NIM EMG® ETT; Medtronic, Inc., Minneapolis, MN, USA) is recommended during thyroid surgery to identify and preserve the integrity of the recurrent laryngeal nerve (RLN) [3,4]. Even though the NIM EMG® ETT is not widely used yet, it can be used not only to identify the RLN during thyroidectomy, but also to monitor the vagus nerve during head and neck surgery [4]. The NIM EMG® ETT is a wire-reinforced endotracheal tube equipped with surface electrodes. The endotracheal tube laryngeal surface electrodes have the following important advantages: ease of setup and use, non-invasive nature and ability to sample greater regions of evoked muscle action potentials; however, any improper connection of wires or tube rotation or displacement of the electrodes could result in equipment failure [3,4]. Such foreign bodies within airway lumen can potentially cause management problems, throughout positioning a fiberoptic instrument [5]. On the other hand, although NIM EMG® ETT is available in various sizes, its outer diameter (OD) is larger than that of the corresponding size of a standard endotracheal tube. This increased OD could be an important limitation to its use in case of nasal intubation of NIM EMG® ETT. Use of Glidescope® has been reported for confirm correct placement of the NIM EMG® ETT [6,7]. We report a case of expected difficult airway management in which a nasal intubation with NIM EMG® ETT was successfully obtained by performing a fiberoptic bronchoscopy.

## Case presentation

**Figure 1:**
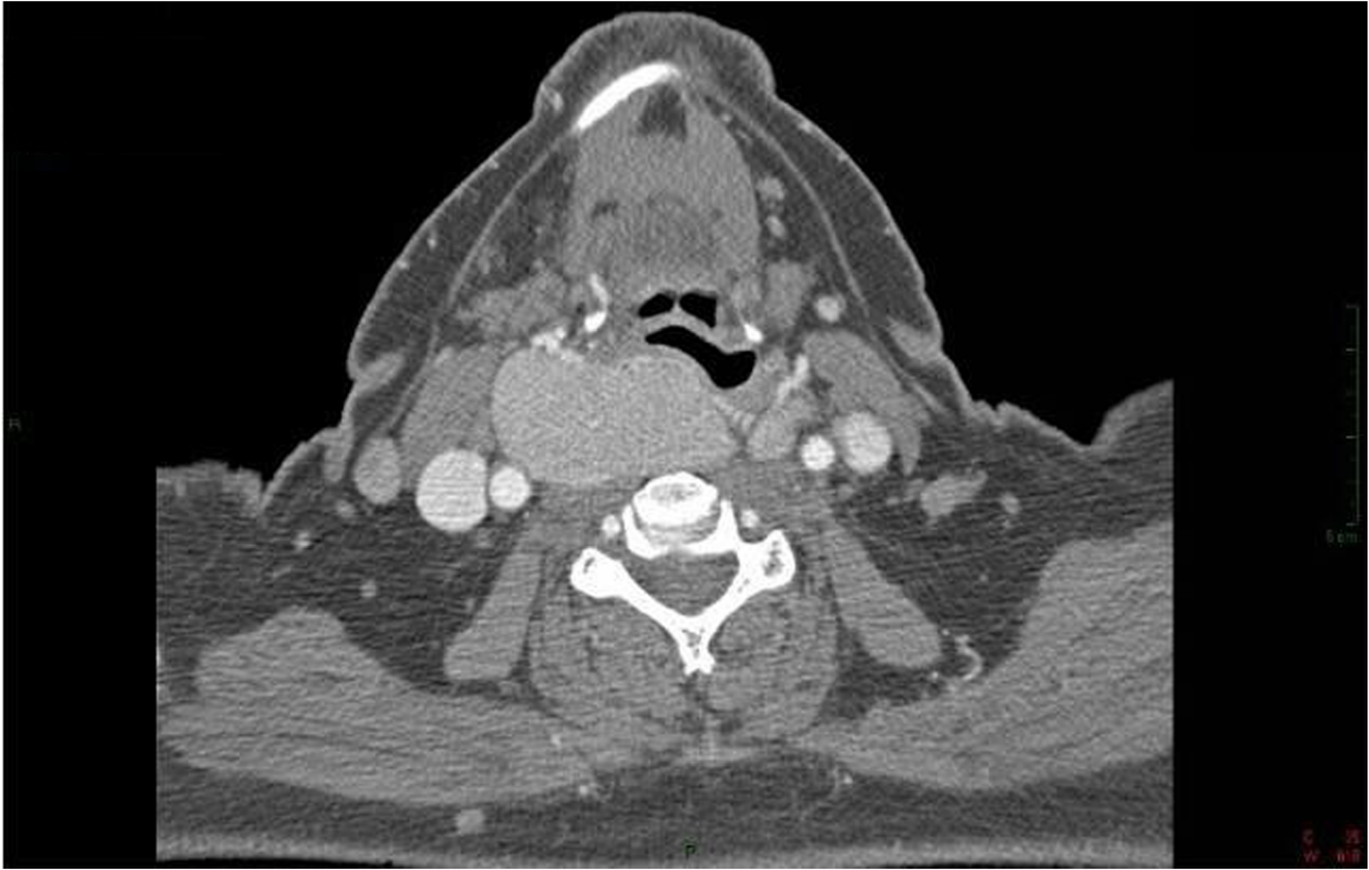
Preoperative computed tomography scan shows the goiter compressing trachea and esophagus.

A 70-year-old woman was scheduled for elective total thyroidectomy due to plunging intrathoracic goiter. The patient had a history of Hashimoto’s thyroiditis. The growth of thyroid had caused over time dysphagia, mild-to-moderate dyspnea and right vocal cord paralysis with mild dysphonia. Other significant medical history included obesity (body mass index 43), arterial hypertension, chronic atrial fibrillation, fibrocystic mastopathy, carotid atheroma, hiatal hernia and pharyngo esophageal diverticulum. Preoperative indirect laryngoscopy, performed by an otolaryngologist, pointed out a massive bombè of the hypopharyngeal wall to the right and hypomobile right vocal cord. The epiglottis was edematous and the glottis could not be identified. On physical examination, the tongue was large and a Mallampati’s score of 3 was determined. Finally, computed tomography scan revealed: 1) an intrathoracic goiter externalization to the right with cranial expansion toward the larynx and pharynx, and an axial deviation of the trachea to the right 2) right vocal cord paralysis in paramedian position 3) thyroid volume estimation of about 110 ml (Figure 1).

Therefore a difficult intubation was expected. Because of the presence of large tongue, right vocal cord paralisys and massive bombè of the hypopharyngeal wall, a nasal fiberoptic intubation was planned. However, since the nasal intubation can be associated with bleeding especially in patients with coagulative disorders, as it was in this case, a NIM EMG® ETT with a small inner diameter was used. Indeed the NIM EMG® ETT outer diameter is larger than the corresponding standard tube [10.2 mm for a 7.0 NIM EMG tube vs. 9.5 mm for a standard 7.0 polyvinyl chloride tube]. Awake fiberoptic intubation was considered to be an appropriate choice because of the patient’s ability to cooperate, according to the American Society of Anestesiologist’s difficult airway algorithm [8]. After topical application of 2% lidocaine to nasal mucosa, we performed fiberoptic bronchoscopy (PentaxR FB-15P, Pentax Corporation, Tokyo, Japan) pre-mounted with a 6.0 mm inner diameter NIM EMG® EET through the nasal cavity, while the patient was spontaneously breathing. Oxygenation was obtained by using a face mask with 100% oxygen. As a consequence, oxygenation was adequate during the procedure. Intubation was performed under light sedation. The patient was premedicated with midazolam (0.03 mg/kg), fentanyl (3 mcg/kg), and propofol (1.5 mg/kg) based on predicted body weight. Prior to insertion, the tube was lubrificated with lidocaine gel. When the fiberscope’s tip was at the carina, the next step was to pass the endotracheal tube and additional lidocaine has been administered through the working channel of the bronchoscope in the trachea. A Cormak grade 2 view of the vocal cords was obtained, the NIM EMG® EET was passed easily and the video component of the fiberoptic allowed tube placement with confirmation on the video screen. For optimal electromyographic recording, the electrodes should be placed exactly between and perpendicular to vocal cords. The use of fiberoptic bronchoscopy allowed a correct visualization of vocal cords and so the optimal placement of NIM EMG® EET. Intubation time was 20 minutes. Anaesthesia was maintained with sevoflurane and fentanyl and the patient was stable for the entire duration of the intervention (240 minutes). Major nasal bleeding was not encountered. The patient was ventilated with an external positive end-expiratory pressure (PEEPe) of 8 cmH_2_O, a tidal volume of 8 ml/kg of predicted body weight and a respiratory rate of 13 breaths/min. The latter has been chosen to maintain normocapnia. At the of end surgery, the patient was taken to the intensive care unit, because of the need of performing extubation in a secure environment. The patient was extubated on the first postoperative day and she had no complications.

## Conclusion

The present case report suggests that the use of fiberoptic bronchoscopy allows correct positioning of the NIM EMG® EET, even if nasal intubation is needed. This approach has never been previously reported and it’s of clinical interest because it allows laryngeal nerve monitoring even in obese patients with expected difficult airway management.

## Consent

Written informed consent was obtained from the patient for publication of this Case Report and its accompanying images. A copy of the written consent is available for review by the Editor-in-Chief of this journal.

## Abbreviations

NIM EMG® ETT: Nerve integrity monitor electromyographic endo-tracheal tube; RLN: Recurrent laryngeal nerve; OD: Outer diameter; PEEPe: External positive end-expiratory pressure.

## Competing interests

The authors declare that they have no competing interests.

## Authors’ contributions

SS looked after the patient and conceived of the idea for the report and wrote the draft. DM wrote the first draft. VC and RR together revised and rewrote the manuscript. VM helped to draft the manuscript and assisted during the procedure. All authors read and approved the final manuscript.
